# The complete mitochondrial genome of *Anoplocnemis curvipes* F. (Coreinea, Coreidae, Heteroptera), a pest of fresh cowpea pods

**DOI:** 10.1080/23802359.2017.1347829

**Published:** 2017-07-18

**Authors:** M. Carmen Valero, James Adebayo Ojo, Weilin Sun, Manuele Tamò, Brad S. Coates, Barry R. Pittendrigh

**Affiliations:** a Carl R. Woese Institute of Genomic Biology, University of Illinois at Urbana-Champaign, IL, USA;; b Department of Crop Production, Kwara State University, Ilorin, Nigeria;; c Department of Entomology, Michigan State University, East Lansing, MI, USA;; d International Institute of Tropical Agriculture, Cotonou, Benin;; e United States Department of Agriculture – Agricultural Research Service, Corn Insect and Crop Genetics Research Unit, Genetics Laboratory, Iowa State University, Ames, USA

**Keywords:** *Anoplocnemis curvipes*, mitochondrial genome, DNA sequence, phylogenetic

## Abstract

The complete 16,345-bp mitochondrial genome of the agriculturally destructive pod sucking pest, the giant coreid bug, *Anoplocnemis curvipes* (Hemiptera: Coreidae), was assembled from paired-end Illumina HiSeq 2500 reads. The *A. curvipes* mitochondrial genome consists of 13 protein coding genes (PCGs), 22 tRNAs, 2 rRNAs and a control region in the order and orientation typical among insects. PCG initiation codons (ATG, ATC, ATT and ATA) with termination codon (TAA) are used with the exception of TAG stop codons by Cytb and ND3. All tRNA genes fold into predicted cloverleaf secondary structures having requisite triplets on the anticodon loop, apart from tRNA-Ser1 (AGN) whose dihydrouridine (DHU) arm forms a simple loop. The phylogenetic analysis of hemipteran mitogenomes clusters to the family level and supports the monophyly of the five superfamilies in Pentatomomorpha of Hemiptera. The Coreoidea and Pyrrhocoroidea are sister groups, while Coreidae and Alydidae are sister groups to Rhopalidae. These analyses provide insight to mitogenomics and evolutionary relationships among pentatomoid insects.

The giant coreid bug, *Anoplocnemis curvipes,* is a pod sucking pest insect of cultivated cowpea that causes damage by sucking sap from green pods, which leads to shrivelling and premature drying of the pod. Despite this destructive feeding behaviour, little genetic information exists for this species. Insect mitochondrial genomes (mitogenomes) are small in size, lack recombination and are maternally inherited and evolutionarily at a rapid rate, which make them an appealing tool for studying population structure, species differentiation and phylogenetics (Cameron 2014; Coates 2014). In the following, we describe the complete mitogenome sequence of *A. curvipes* and provide molecular and phylogenetic information for studies on Pentatomomorpha of Hemiptera.

Total genomic DNA extracted from a single adult *A. curvipes* collected from Benin (N 10°58.227 E 003°14.550), from which Illumina HiSeq2500 data were obtained as described by Coates (2014). Sequence data were assembled using CLC Genomics Workbench 8.5 (Qiagen, Valencia, CA) and annotated as described by Sun et al. (2017), and a phylogeny for the infraorder Pentatomomorpha of Hemiptera was constructed with MEGA 7.014 (Kumar et al. 2016) as described previously (Sun et al. 2017). The resulting complete 16,345-bp mitogenome (GenBank accession KY906099) has a high A + T nucleotide content (41.5% A, 31.3% T, 16.6% C and 10.5% G), but is similar to that obtained in other hemipteran mitogenomes (Hua et al. 2008; Sun et al. 2017). The encoded 13 PCGs, 22 tRNA genes and 2 rRNA genes, and a non-coding A + T-rich region are in an order and orientation that is also shared with the ancestral animal mitogenome (Boore 1999) and among other hemipteran insects (Hua et al. 2008; Sun et al. 2017). ATP 8 and ATP 6, and ND4 and ND4L each overlap one another by seven nucleotides. The PCGs of *A. curvipes* mitogenome are initiated with ATN start codon (ATG for ND2, ATP 6, COX III, ND5, ND4 and Cytb; ATC for COX I, ATP8 and ND3; ATT for COX II, ND4L and ND1; and ATA for ND6), and termination codons are comprised of 2 TAG and 11 TAA.

The alignment of 13 concatenated derived amino acid sequences from 26 complete hemipteran mitogenomes within the infraorder Pentatomomorpha, which includes 5 Coreoidea, 2 Pyrrhocoroidea, 5 Lygaeoidea, 9 Pentatomoidea and 5 Aradoidea (GenBank accessions provided in Figure 1). The resulting maximum likelihood (ML) analysis predicted that Coreoidea and Pyrrhocoroidea form sister groups and each superfamily of Pentatomorpha (Coreoidea, Pyrrhocoroidea, Lygaeoidea, Pentatomoidea and Aradoidea) were all monophyletic with high node support. These results agree with prior morphological and molecular phylogenetic analyses that showed Coreoidea and Pyrrhocoroidea as sister groups (Tian et al. 2011; Kocher et al. 2014), as well as the monophyly of the five superfamilies in Pentatomomorpha (Hua et al. 2008). Our analyses comparatively expanded the number of mitogenomes from 15 to 26 Heteroptera. Our analysis also suggests that Coreidae and Alydidae are sister groups to Rhopalidae, which agrees with prior predictions by Xie et al. (2005) and Hua et al. (2008).

**Figure 1.**
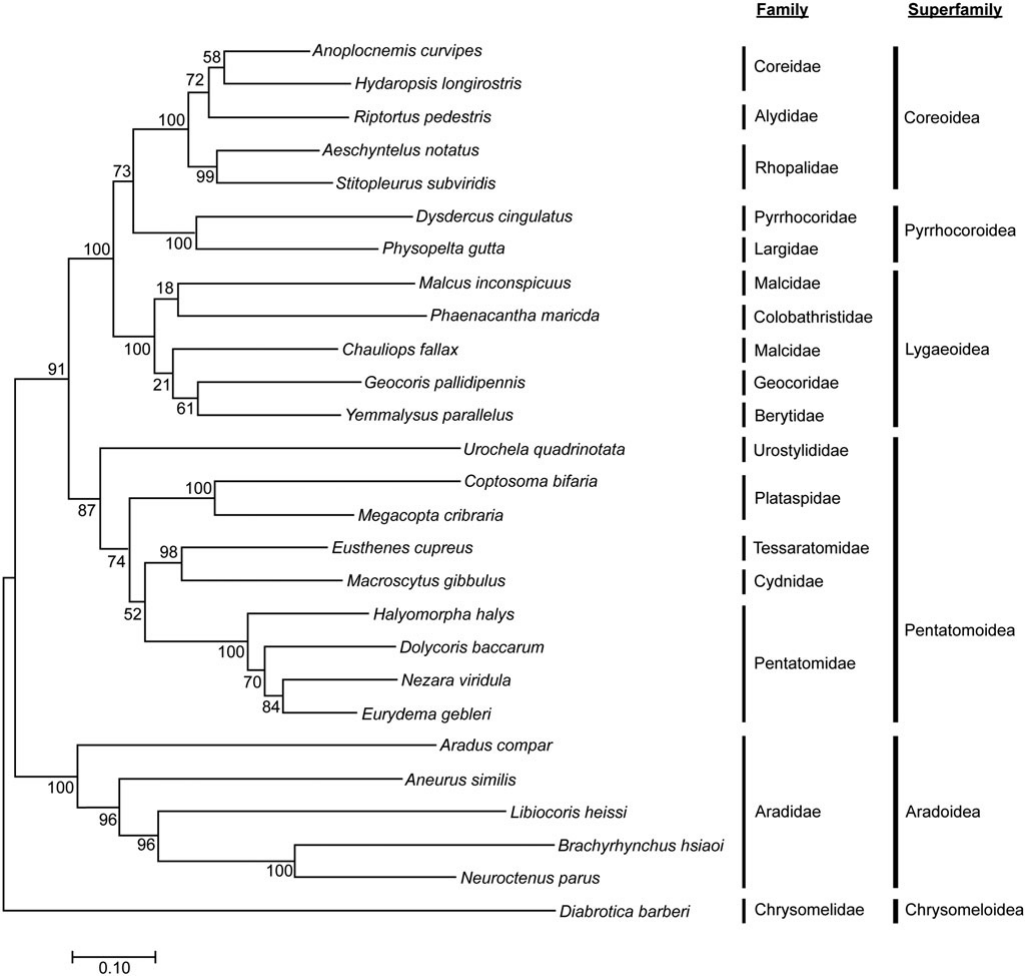
The maximum likelihood estimation of phylogenetic relationships among sites from the insect infraorder Pentatomomorpha based on 13 protein coding genes from complete or near-complete mitochondrial genomes. *D. barberi* (Coleoptera: Chrysomelidae) was used to root the tree as an out-group. The accession numbers are as follows: *H. longirostris* (EU427337); *R. pedestris* (EU427344); *A. notatus* (EU427333); *S. subviridis* (EU826088); *D. cingulatus* (EU427335); *P. gutta* (EU427343); *P. marcida* (EU427342); *G. pallidipennis* (EU427336); *Y. parallelus* (EU427346); *C. fallax* (JX839706); *M. inconspicuus* (EU427339); *U. quadrinotata* (JQ743678); *C. bifaria* (EU427334); *M. cribraria* (JF288758); *E. cupreus* (JQ910983); *M. gibbulus* (EU427338); *H. halys* (FJ685650); *D. baccarum* (KC460537); *N. viridula* (EF208087); *E. gebleri* (KP207595); *A. compar* (JQ780818); *A. similis* (JQ780816); *L. heissi* (JQ780819); *B. hsiaoi* (HQ441232); *N. parus* (EU427340); and *D. barberi* (KF669870).

## References

[CIT0001] Boore JL. 1999 Animal mitochondrial genomes. Nucleic Acids Res. 27:1767–1780. 10101183 10.1093/nar/27.8.1767 PMC148383

[CIT0002] Cameron SL. 2014 Insect mitochondrial genomics: implications for evolution and phylogeny. Annu Rev Entomol. 59:95–117. 24160435 10.1146/annurev-ento-011613-162007

[CIT0003] Coates BS. 2014 Assembly and annotation of full mitochondrial genomes for the corn rootworm species, *Diabrotica virgifera virgifera* and *Diabrotica barberi* (Insecta: Coleoptera: Chrysomelidae), using Next Generation Sequence data. Gene. 542:190–197. 24657060 10.1016/j.gene.2014.03.035

[CIT0004] Hua J, Li M, Dong P, Cui Y, Xie Q, Bu W. 2008 Comparative and phylogenomic studies on the mitochondrial genomes of Pentatomomorpha (Insecta: Hemiptera: Heteroptera). BMC Genomics. 9:610. 19091056 10.1186/1471-2164-9-610 PMC2651891

[CIT0005] Kocher A, Kamilari M, Lhuillier E, Coissac E, Péneau J, Chave J, Murienne J. 2014 Shotgun assembly of the assassin bug *Brontostoma colossus* mitochondrial genome (Heteroptera, Reduviidae). Gene. 552:184–194. 25240790 10.1016/j.gene.2014.09.033

[CIT0006] Kumar S, Stecher G, Tamura K. 2016 MEGA7: Molecular Evolutionary Genetics Analysis version 7.0 for bigger datasets. Mol Biol Evol. 33:1870–1874. 27004904 10.1093/molbev/msw054 PMC8210823

[CIT0007] Sun W, Huynh BL, Ojo JA, Coates BS, Kusi F, Roberts PA, Pittendrigh BR. 2017 Comparison of complete mitochondrial DNA sequences between old and new world strains of the cowpea aphid, *Aphis craccivora* (Hemiptera: Aphididae). Agri Gene. 4:23–29.

[CIT0008] Tian X, Xie Q, Li M, Gao C, Cui Y, Xi L, Bu W. 2011 Phylogeny of pentatomomorphan bugs (Hemiptera-Heteroptera: Pentatomomorpha) based on six Hox gene fragments. Zootaxa. 2888:57–68.

[CIT0009] Xie Q, Bu W, Zheng L. 2005 The Bayesian phylogenetic analysis of the 18S rRNA sequences from the main lineages of Trichophora (Insecta: Heteroptera: Pentatomomorpha). Mol Phylogenet Evol. 34:448–451. 15619455 10.1016/j.ympev.2004.10.015

